# Evaluating negative-pressure wound therapy after abdominoperineal resection: a systematic review of efficacy and technical variability

**DOI:** 10.1007/s10151-025-03212-5

**Published:** 2025-09-23

**Authors:** A. Litchinko, F. Ris, B. Noiret, M. Adamina, Q. Denost

**Affiliations:** 1 Division of Digestive Surgery, HFR Fribourg – Cantonal Hospital (University Hospitals of Fribourg), Chemin des Pensionnat, 2/6, 1752 Fribourg, Switzerland; 2 Faculty of Science, HFR Fribourg – Cantonal Hospital, 1711 Fribourg, Switzerland; 3 Division of Digestive Surgery, University Hospitals of Geneva, Rue Gabrielle-Perret-Gentil 4, 1211 Geneva 14, Switzerland; 4 Bordeaux Colorectal Institute, 220 Rue Mandron, 33300 Bordeaux, France

**Keywords:** Abdominoperineal resection, Prophylactic negative-pressure wound therapy, Perineal wound complications, Surgical site infection

## Abstract

**Background:**

Perineal wound complications after abdominoperineal resection (APR) for anal or low rectal cancer remain a significant clinical concern, frequently leading to surgical site infections (SSIs), wound dehiscence, and delayed healing. These complications contribute to increased patient morbidity, prolonged hospitalization, and higher healthcare costs. Prophylactic negative pressure wound therapy (pNPWT) has been proposed to improve wound outcomes in this context, but evidence regarding its effectiveness remains inconclusive.

**Objective:**

This systematic review evaluates the clinical outcomes and technical application of pNPWT in closed perineal wounds following APR, with a focus on its potential impact on SSIs, wound dehiscence, and healing time.

**Methods:**

A systematic literature search was conducted in MEDLINE, Embase, and the Cochrane Library in accordance with PRISMA guidelines. Eligible studies included randomized controlled trials and observational studies assessing pNPWT after APR. Key outcomes of interest were SSI rates, wound dehiscence, healing time, and length of hospital stay. Due to clinical and methodological heterogeneity, a narrative synthesis was performed.

**Results:**

In total, eight studies met the inclusion criteria. The results were heterogeneous: while three studies reported reduced SSI rates with pNPWT compared with conventional wound management, two studies observed higher SSI rates in the pNPWT groups. Variability in device type (canister-based versus portable systems), negative pressure settings, application duration, and patient selection limited the comparability across studies. The risk of bias was moderate to high in several studies, and outcome reporting was inconsistent.

**Conclusions:**

Current evidence does not allow for definitive conclusions regarding the clinical benefit of pNPWT after APR. While some studies suggest potential advantages, particularly in terms of SSI reduction, results remain inconsistent and device-dependent. Further high-quality randomized trials are required to clarify the role of pNPWT and to define optimal application protocols in this challenging surgical context.

**Supplementary Information:**

The online version contains supplementary material available at 10.1007/s10151-025-03212-5.

## Introduction

Low rectal cancer, defined as tumors located within 5 cm of the anal verge and anal cancers, presents unique challenges in surgical management due to their proximity to critical anatomical structures such as the anal sphincters, pelvic nerves, and vasculature [1–5]. Achieving oncological control while preserving sphincter function is often difficult, particularly for tumors that invade or closely approach the external sphincter. Abdominoperineal resection (APR) is the standard surgical approach for such cases, particularly when sphincter preservation is not feasible or when functional outcomes would be poor [6–8]. Initially described by Miles more than a century ago, APR involves en bloc resection of the distal colon, rectum, anus, and the surrounding internal and external sphincters, culminating in the creation of a permanent colostomy [9]. Despite advances in surgical techniques, APR is associated with significant postoperative morbidity, particularly related to perineal wound complications [10–12].

Perineal wound complications following APR remain a major source of morbidity. These complications include surgical site infections (SSIs), wound dehiscence, perineal herniation, chronic sinus formation, and delayed wound healing [10, 13]. Such complications have a profound impact on patient outcomes, leading to prolonged hospitalization, frequent reoperations, delayed return to normal activities, and increased healthcare costs [14–16]. Studies have shown that perineal wound complications occur in up to 50% of patients undergoing APR, with factors such as preoperative radiotherapy, extensive resections, comorbidities such as diabetes or obesity, and poor nutritional status contributing to this high incidence [10, 17, 18]. Additionally, these complications significantly affect the patient’s quality of life, often resulting in chronic pain, discomfort, and functional limitations [19–21].

The extensive nature of APR, particularly in cases requiring extralevator abdominoperineal excision (ELAPE) or multivisceral resection for advanced or recurrent tumors, often leaves a large perineal defect. Traditional methods of closure include suturing the levator ani muscles, using synthetic or biological meshes, or employing flap reconstructions such as the pedicled vertical rectus abdominis myocutaneous (VRAM) flap, gracilis muscle flap, or gluteal artery perforator flap [22–25]. However, even with these techniques, wound healing is frequently compromised, particularly in patients who have undergone neoadjuvant chemoradiotherapy. Radiotherapy, a cornerstone of treatment for locally advanced rectal cancer and anal cancer, induces significant tissue changes, including fibrosis, hypoxia, and impaired angiogenesis, all of which contribute to delayed or incomplete wound healing. In fact, patients who receive preoperative radiotherapy are nearly twice as likely to experience wound complications compared with those who do not [18, 26, 27].

Treatment landscape for low rectal cancer has shifted toward total neoadjuvant therapy, which integrates both chemoradiotherapy (CRT) and systemic chemotherapy prior to surgery [28]. This approach has been shown to increase rates of tumor downstaging and complete pathological response, improving overall survival and reducing local recurrence. However, while TNT offers oncological benefits, it also exacerbates the risk of postoperative wound complications, particularly in patients undergoing APR. Chemoradiotherapy disrupts normal wound healing processes by inducing tissue hypoxia, reducing cellular proliferation, and increasing fibrosis, while systemic chemotherapy further compromises immune function and delays tissue regeneration.

Given the high rate of wound complications, the application of advanced wound management strategies has become increasingly important. Negative pressure wound therapy (NPWT), initially developed for open wounds and delayed healing, has been widely adopted for use in perineal wounds following APR [29, 30]. The mechanism of NPWT involves applying subatmospheric pressure to the wound bed, promoting granulation tissue formation, enhancing perfusion, and reducing bacterial load. Traditionally, NPWT has been used to manage open perineal wounds or in cases where primary closure has failed, facilitating healing by secondary intention. However, more recent innovations have focused on the preventive application of NPWT on closed surgical incisions, referred to as prophylactic NPWT (pNPWT) or incisional NPWT (iNPWT). NPWT devices can be broadly categorized into canister-based systems (e.g., VAC^®^), which deliver higher negative pressures (typically around −125 mmHg), and portable canisterless systems (e.g., PICO^®^ and Avelle^®^), which operate at lower pressures (−70 to −80 mmHg) and collect exudate within the dressing itself. The choice of device, pressure setting, and duration of application vary significantly between studies and may influence outcomes.

Prophylactic NPWT has shown promise in reducing the incidence of SSIs and wound dehiscence across various surgical fields, including gastrointestinal, colorectal, and vascular surgery. Studies have reported that pNPWT can significantly lower the risk of SSIs, particularly in high-risk surgical sites such as the perineum. In the context of APR, where perineal wounds are particularly prone to complications due to the large defect size, anatomical location, and radiotherapy-induced tissue damage, pNPWT represents a potential strategy for improving wound outcomes. Early studies examining the use of pNPWT in APR have demonstrated encouraging results, with reductions in SSI rates and improvements in wound-healing times. However, despite these positive findings, pooled evidence remains scarce, and questions remain regarding the long-term efficacy, cost-effectiveness, and optimal timing of pNPWT application. The integration of pNPWT into standardized postoperative protocols for patients undergoing APR is not yet well-established. Additionally, results across literature are inconsistent, and several studies have even reported increased rates of SSI in the pNPWT group. Understanding the specific patient populations most likely to benefit from pNPWT—such as those with high BMI, preoperative radiotherapy, or extensive resection—will be critical for tailoring its use. Additionally, considerations regarding healthcare costs, resource allocation, and patient adherence to postoperative wound care protocols will also need to be addressed. As such, further research is required to determine the most effective implementation strategies for pNPWT, as well as to quantify its impact on patient outcomes in both the short and long term.

The primary objective of this systematic review is to provide a comprehensive assessment of the incidence, types, and management of wound complications following APR, with a particular focus on the role of pNPWT in preventing wound-related complications such as SSIs and wound dehiscence. This review also aims to describe the variability in pNPWT application protocols and device types reported across studies and to evaluate whether this heterogeneity may partly explain the inconsistent clinical outcomes.

## Materials and methods

This systematic review follows the Preferred Reporting Items for Systematic Reviews and Meta-analyses (PRISMA) guidelines to ensure methodological transparency and consistency [31]. The study was preregistered in the International Prospective Register of Systematic Reviews (PROSPERO) number CRD42025598632.

### Eligibility criteria

The review included original studies published in English that examined the use of prophylactic negative-pressure wound therapy (pNPWT) for the prevention of wound-related complications in closed perineal wounds following abdominoperineal resection (APR). Eligible study types included randomized controlled trials (RCTs), cohort studies, case series, and relevant conference abstracts. Exclusion criteria were studies involving patients under 18 years of age, secondary analyses of previously published data, and studies lacking sufficient data for extraction. “Sufficient data” was defined as reporting at least one primary outcome of interest, namely surgical site infections (SSIs), wound dehiscence, healing time, or length of hospital stay, with extractable numerical or comparative results. Studies involving nonmalignant indications were not excluded if APR was performed and perineal wound outcomes were available. However, two studies were excluded during screening for focusing solely on non-oncologic surgical contexts.

### Search strategy

A comprehensive search was conducted across MEDLINE, Embase, and the Cochrane Library from their inception to September 2024. The search strategy was developed using a combination of free-text terms and index terms (e.g., MeSH in MEDLINE and Emtree in Embase) tailored to capture relevant studies on pNPWT and APR. Boolean operators (AND, OR, NOT) were used to refine the search results, ensuring coverage of relevant literature. The detailed search strategy is outlined in Table 1. Additional records were identified by manually searching the reference lists of included studies. Clinical trial registries, including ClinicalTrials.gov and the WHO International Clinical Trials Registry Platform, were also searched to identify relevant ongoing or unpublished studies.

### Study selection

All retrieved studies were independently screened by two reviewers (A.L. and Q.T.) on the basis of titles and abstracts. Full texts of potentially eligible studies were then assessed for inclusion according to predefined criteria. Discrepancies in study selection were resolved by discussion with a third reviewer (M.A.). A PRISMA flow diagram was used to depict the study selection process, from initial search results to final inclusion [31].

### Data extraction

Data from the included studies were extracted using a standardized form. The extracted data included study design, population characteristics, intervention details (pNPWT), comparator groups, and outcomes such as surgical site infections (SSIs) and wound dehiscence. Additional extracted variables included the type of NPWT device (e.g., canister-based VAC^®^ or portable PICO^®^/Avelle^®^ systems), the pressure applied, duration of therapy, and the method of perineal wound closure (e.g., direct suture, flap, or mesh reconstruction) if available in the studies. Two reviewers (A.L. and Q.T.) independently performed data extraction, with any discrepancies resolved through consensus or consultation with a third reviewer (FR). Where available, raw data were included for further analysis.

### Risk of bias assessment

The quality of the included studies was assessed using the Newcastle–Ottawa Scale (NOS), specifically the three-domain version (“Selection–Comparability–Outcome”) adapted for nonrandomized cohort and case–control studies in surgical research. This tool was chosen for its applicability to observational designs and allows a maximum score of nine points. Risk of bias was evaluated independently by two reviewers (A.L. and Q.T.) after full-text data reextraction, as per reviewer recommendation. Discrepancies were resolved through discussion with a third reviewer (F.R.). Each study was categorized as having a low, moderate, or high risk of bias on the basis of its total score. A summary of the individual domain scores is provided in Table 3, and the detailed scoring matrix is available in the Supplementary Material.

### Data synthesis and analysis

A narrative synthesis was performed owing to the heterogeneity in study designs, patient populations, and outcome measures.

## Results

### Study selection

**Fig. 1:**
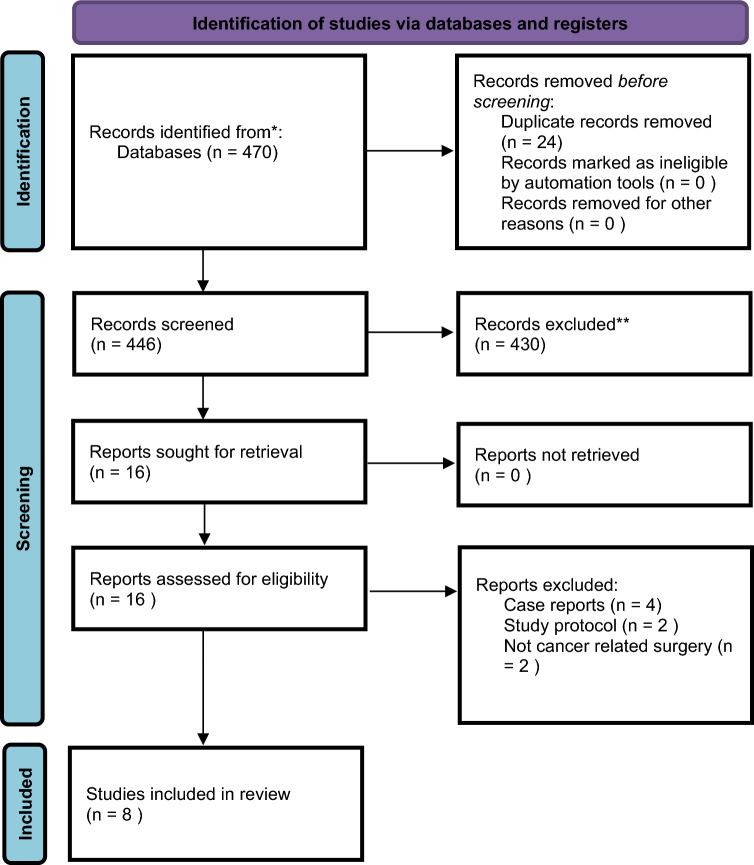
PRISMA flowchart

The database search retrieved a total of 470 studies from MEDLINE, Embase, Web of Science, and the Cochrane Library. After removing 24 duplicates and irrelevant studies, 446 studies were screened on the basis of their titles and abstracts. Following a full-text assessment of 16 reports, 8 studies met the inclusion criteria and were included in the final analysis. The study selection process is detailed in Fig. 1 (PRISMA flow diagram). In total, two studies were excluded during full-text review because they involved APR performed exclusively for benign disease, while the included studies either focused on malignancy or had mixed populations.

### Study characteristics

**Fig. 2:**
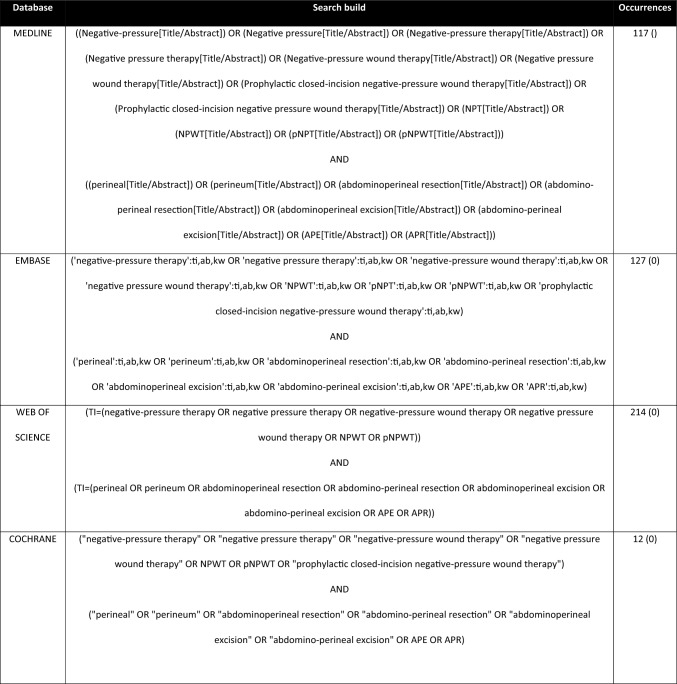
Literature search strategy

A total of eight studies met the inclusion criteria for this systematic review, examining the impact of prophylactic negative-pressure wound therapy (pNPWT) on perineal wound-related complications following abdominoperineal resection (APR). These included a variety of study designs—cohort studies, retrospective analyses, and pilot trials—with sample sizes ranging from 6 to 146 patients. Importantly, none of these studies were randomized controlled trials. Additionally, different types of pNPWT devices were employed across the included studies (KCI, Smith and Nephew, ConvaTech and 3M™), with different ranges of negative pressure from −40 to −125 mmHg. Device types included both canister-based systems (e.g., VAC^®^) and portable canisterless systems (e.g., PICO^®^). Pressure settings and application duration varied, ranging from 5 to 12 days depending on institutional protocols (Fig. 2).

### Surgical site infections (SSI)

All studies reported on the incidence of surgical site infections (SSIs), a major complication following APR. Chadi et al. [32] and Chung et al. [33] both observed significantly lower SSI rates in the pNPWT groups compared with standard dressing groups. Specifically, Chadi et al. [32] reported an SSI rate of 14.8% in the pNPWT group compared with 40.6% in the control group, a risk reduction of over 25%. Similarly, Chung et al. [33] found a 9.1% SSI rate in the pNPWT group compared with 41.7% in the control group, further supporting the efficacy of pNPWT in reducing SSIs.

However, not all studies demonstrated such significant differences. Van der Walk et al. [30] reported high complications rates in both the pNPWT group (70%) and the standard dressing group (60%), with no statistically significant difference between the two groups. In this study, pNPWT did not reduce wound complications, but wound infections occurred slightly later and appeared to have a less severe clinical course. Additionally, the duration of wound healing was shorter after treatment with pNPWT. Rather et al. [34] also found similar SSI rates in both groups, with 50% in the pNPWT group and 64.3% in the control group. Although these differences were not statistically significant, the authors noted that the infections in the pNPWT group were less severe, and fewer patients required secondary interventions. In studies using portable systems such as PICO^®^, no reduction in SSI rates was observed, and problems with adherence were frequently reported.

### Wound dehiscence

Wound dehiscence, another critical complication post-APR, was also evaluated in multiple studies. Sumrien et al. [35] found a significant reduction in wound dehiscence with pNPWT, reporting a rate of 9.4% compared with 40% in the standard dressing group, demonstrating the potential for pNPWT to enhance perineal wound healing. By contrast, Salmenkylä et al. [36] found significant differences in wound dehiscence rates between the two groups, reporting rates of 10% for pNPWT and 5% for control group, with a study population of 21 patients. This study also highlighted device-related complications, with 62% of the pNPWT devices failing due to vacuum failure or device-related issues, which likely contributed to the lack of significant difference in outcomes. An additional factor that may influence wound healing outcomes is the use of perineal drainage, which was not consistently reported across studies. Differences in drainage protocols, including the type, duration, and indication for perineal drains, could impact the interpretation of wound healing results and contribute to variability between studies. Moreover, the use of biological or synthetic meshes to reinforce the perineal closure, particularly in patients receiving pNPWT, remains an important yet underreported variable. While several studies mention mesh reinforcement, they do not systematically analyze its impact on wound-healing outcomes or device performance. Further research is needed to clarify the role of these adjunctive measures in optimizing perineal wound management post-APR. Closure technique (e.g., flap versus primary closure) was inconsistently reported and may also influence wound outcomes, but no subgroup analysis was performed.

### Other complications

Several studies reported on other complications, such as intraabdominal abscesses, emergency department (ED) visits, and reoperations. Chadi et al. [32] reported an intraabdominal abscess rate of 7.4% in the pNPWT group compared with 3.1% in the control group, though this difference was not statistically significant. Similarly, Rather et al. [34] found a 18.8% need for secondary VAC therapy in the pNPWT group, compared with 50% in the standard dressing group, suggesting that pNPWT may reduce the need for more aggressive wound interventions. Timing of complications relative to device removal was not systematically analyzed. In most studies, infections occurred after device removal, but precise chronology was often lacking.

### Length of hospital stay

Only one study, Salmenkylä et al. [36], reported on the length of hospital stay. The pNPWT group had a slightly longer mean hospital stay of 15 days, compared with 13 days for the control group. This was attributed to device failure and subsequent delays in wound healing management. However, without more studies reporting on this outcome, it is difficult to draw strong conclusions about the impact of pNPWT on length of stay. Given the limited data and the presence of confounders such as infection severity and reinterventions, this parameter remains difficult to interpret.

### Device-related complications

The use of pNPWT was generally well-tolerated across the studies, but a few noted technical challenges. Salmenkylä et al. [36] reported that 62% of pNPWT devices malfunctioned due to vacuum failure or poor adhesion, necessitating premature device removal. These technical difficulties likely contributed to the lack of significant differences in wound-related complications in this study. Van der Walk et al. [30] also noted instances of device dysfunction, though the frequency and impact on patient outcomes were not thoroughly quantified. Most groups used a prophylactic NPWT for a minimum of 5–7 days with a negative pressure from −40 to −125 mmHg. Various devices were used from superficial and portable motors (PICO^®^, Smith and Nephew) to standard vacuum device (VAC^®^, KCI). None of the included studies systematically compared outcomes between device types or pressure levels, nor did they analyze adherence issues by device brand.

### Comorbidities and preoperative radiation

Several studies provided data on comorbidities and the use of preoperative radiation therapy, both of which can influence wound healing. Chadi et al. [32] reported that 45% of patients in their study received preoperative radiation therapy, while 15% had diabetes. However, no studies directly stratified outcomes on the basis of these factors, limiting the ability to determine their influence on the efficacy of pNPWT. Most indications were rectal cancers with neoadjuvant treatment with radiochemotherapy. None of the studies analyzed a subgroup of patients regarding the dose of radiation, type of tumor (rectal or anal cancer), or wound related complications. This lack of stratification by comorbidity or oncologic subtype represents a limitation in evaluating pNPWT’s effectiveness in specific risk groups.

### Risk of bias

The risk of bias across the included studies was moderate to high, primarily due to limitations in study design, small sample sizes, and lack of randomization. Most studies were observational in nature, with several employing retrospective or cohort designs, which introduces potential selection bias. Only a few studies reported clearly defined methods for patient selection, and many did not adequately account for potential confounding factors such as comorbidities (e.g., diabetes, obesity) or preoperative radiation therapy, both of which are known to influence wound healing outcomes. Blinding was generally not reported or implemented, especially in the context of outcome assessment, leading to a possible detection bias, particularly in subjective outcomes such as surgical site infections (SSIs) and wound healing. In several studies, there was a reliance on historical controls rather than contemporaneous control groups, which may have introduced further bias. For example, patients receiving pNPWT were often compared with control groups from earlier time periods, raising concerns about performance bias, as surgical techniques and postoperative care could have evolved over time.

Furthermore, several studies had small sample sizes, which limited their statistical power and increased the likelihood of type II errors. This was particularly evident in pilot studies where the number of participants was insufficient to draw robust conclusions about the efficacy of pNPWT. Reporting bias may also be a concern, as not all studies consistently reported important outcomes, such as length of hospital stay or device-related complications, which are crucial for understanding the full scope of pNPWT's effectiveness. Additionally, none of the studies evaluated or reported interrater reliability in outcome assessment.

Device-related issues, such as vacuum failure or poor adhesion, were inconsistently reported, raising the possibility of attrition bias if patients with device malfunctions were not adequately followed up or accounted for in the analysis. Finally, the lack of standardization in the intervention (i.e., differences in the type of pNPWT devices, pressure settings, and duration of therapy) across studies may further compromise the internal validity of the findings.

## Discussion

In this systematic review, we analyzed eight studies that examined the effectiveness of prophylactic negative-pressure wound therapy (pNPWT) in reducing perineal wound complications after abdominoperineal resection (APR). While the evidence remains limited by small sample sizes and heterogeneity in study designs, several findings suggest that pNPWT may offer benefits for this high-risk patient population. Among the included studies, multiple demonstrated a significant reduction in the incidence of surgical site infections (SSIs) in patients treated with pNPWT compared with standard wound dressing. The reductions in SSIs ranged from 25% to 30%, with notable results in studies by Chadi et al. [32] and Chung et al. [33], where pNPWT significantly lowered the infection rates compared with controls. In addition, while some studies did not find a statistically significant difference in SSI incidence, they did report that infections in the pNPWT group tended to be less severe and easier to manage. This suggests that even when pNPWT does not prevent infections entirely, it may still reduce the severity of complications, facilitating improved wound management and patient recovery. Wound dehiscence, a critical postoperative complication following APR, was also reduced in patients receiving pNPWT. For instance, Sumrien et al. [35] reported a 9.4% incidence of wound dehiscence in the pNPWT group, compared with 40% in the standard dressing group, a statistically significant improvement. These results suggest that pNPWT not only mitigates the risk of infection but also may improve wound integrity and promote faster closure, reducing the overall burden of postoperative wound management.

However, the overall findings remain inconsistent. Studies using newer portable systems such as PICO^®^ and Avelle^®^ reported no benefit in terms of SSI or dehiscence, and several observed higher infection rates in the pNPWT group. Furthermore, these devices were associated with frequent technical failures, including poor adhesion and vacuum loss, compromising their effectiveness in the perineal region.

Only three of the eight studies showed a statistically significant reduction in SSI, and the others reported neutral or contradictory results. This heterogeneity of findings highlights the need for caution when interpreting pooled effects and underscores the importance of device type, pressure setting, and duration of application in clinical outcomes.

It is crucial to acknowledge the limitations inherent in the studies reviewed. Many were small-scale pilot studies with significant variability in study design, patient populations, and pNPWT application protocols (e.g., pressure settings, duration of therapy, and device types). Device-related issues were also frequently reported; for example, Salmenkylä et al. [13] noted that 62% of pNPWT devices malfunctioned due to poor adhesion or vacuum failure, potentially compromising the therapy’s effectiveness. These technical issues highlight the need for further refinement of pNPWT devices to improve reliability, especially in the challenging perineal region where achieving a secure vacuum seal can be difficult. In terms of study power, the studies with larger sample sizes tended to produce the most promising results, with significant reductions in both SSIs and wound dehiscence. This raises the possibility of type II errors in smaller studies, where a lack of statistical power may have prevented the detection of meaningful differences between pNPWT and standard dressing groups. Consequently, the absence of statistically significant results in these smaller trials does not necessarily negate the potential efficacy of pNPWT.

It is also important to note that none of the included studies conducted subgroup analyses based on known risk factors such as diabetes, obesity, neoadjuvant radiation, or flap closure. The inability to stratify outcomes by these variables limits the generalizability of the findings and prevents identification of patient subgroups most likely to benefit from pNPWT.

The prevention of perineal wound complications following APR is of considerable clinical importance. Such complications not only prolong hospital stays but also increase the risk of secondary infections, thromboembolic events, and nosocomial infections. Additionally, complications can delay the initiation of adjuvant therapies, potentially affecting long-term oncological outcomes. For instance, patients experiencing wound healing delays may be unable to start postoperative chemotherapy or radiation therapy on schedule, which can have detrimental effects on cancer prognosis. Moreover, the economic implications of wound-related complications are significant, with increased healthcare costs associated with prolonged hospital stays, reoperations, and the need for intensive wound management. Even modest improvements in the rates of SSIs and wound dehiscence using pNPWT could result in substantial reductions in healthcare expenditures and improve overall patient outcomes, including faster recovery times and reduced morbidity.

Given these considerations, there is a clear need for large-scale, well-designed randomized controlled trials (RCTs) to provide more definitive evidence on the efficacy of pNPWT in preventing perineal wound complications after APR. Future studies should focus on standardizing protocols, including consistent pressure settings and duration of therapy, as well as ensuring patient diversity to explore the effectiveness of pNPWT across various risk groups (e.g., patients with comorbidities such as diabetes or those receiving preoperative radiation therapy). Studies should also directly compare different device types and pressure levels, and report adherence issues, timing of complications, and long-term wound and oncological outcomes. Moreover, the long-term benefits of pNPWT, including its impact on oncological outcomes and quality of life, should be examined to fully understand its potential clinical value. While several studies suggest that pNPWT may reduce SSIs and wound dehiscence in selected patients undergoing APR, the current evidence remains insufficient to support routine use in all settings. A tailored approach based on patient risk profile and institutional capabilities is likely warranted.

Further investigation is required to confirm its efficacy and refine its application. Given the high incidence of perineal wound complications, even modest improvements with pNPWT could lead to meaningful clinical and economic benefits.

## Conclusions

This review underscores the potential of prophylactic negative-pressure wound therapy (pNPWT) to significantly reduce surgical site infections (SSIs) and enhance wound healing in patients undergoing abdominoperineal resection (APR). However, only a minority of studies showed a statistically significant benefit, and conflicting findings remain common. Large-scale studies have demonstrated substantial reductions in SSI rates and overall wound complications with pNPWT compared with conventional dressings. Furthermore, its association with decreased wound dehiscence suggests broader benefits for maintaining wound integrity and expediting recovery. Despite these promising findings, the current body of evidence is constrained by considerable heterogeneity in patient populations, surgical techniques, and pNPWT protocols, complicating direct comparisons across studies. Additional challenges stem from small sample sizes, inconsistent control groups, and technical limitations such as vacuum device malfunctions, which highlight the need for both standardization and device optimization. Furthermore, none of the included studies reported stratified outcomes based on patient risk profiles, closure technique, or timing of complications, limiting the granularity of the available evidence. Nevertheless, the favorable outcomes observed in larger, more robust studies suggest that pNPWT holds significant clinical promise, particularly for mitigating wound-related complications in high-risk patients. To fully establish its role in standard surgical practice, larger, high-quality randomized trials are imperative. These studies should focus on protocol standardization, device innovation, and long-term outcomes, including cost-effectiveness analyses. Comparative studies between different NPWT systems and pressure settings are also needed to guide evidence-based clinical choices. In conclusion, while further evidence is required to confirm its widespread applicability, pNPWT has the potential to transform care for patients undergoing APR, offering a pathway to faster recovery, improved surgical outcomes, and reduced healthcare costs in this vulnerable population.

## Supplementary Information

Below is the link to the electronic supplementary material.

Supplementary file1 (DOCX 22 KB)

Supplementary file2 (DOCX 21 KB)

Supplementary file3 (DOCX 23 KB)

## Data Availability

No datasets were generated or analyzed during the current study.
