# Supplementary material for: Evaluating negative-pressure wound therapy after abdominoperineal resection: a systematic review of efficacy and technical variability
Source: Tech Coloproctol. 2025 Sep 23;29(1):168. doi: 10.1007/s10151-025-03212-5 (PMC12457546; doi:10.1007/s10151-025-03212-5)
Supplement: Supplementary file 1 — Supplementary file1 (DOCX 22 KB) [file 10151_2025_3212_MOESM1_ESM.docx]

Table 1. Study Designs, Interventions and Key Outcomes in pNPWT

| Authors (date) & *name* | Study Design & population | Sample Size | pNPWT type, negative pressure used and duration | Outcomes | Key Findings |
| --- | --- | --- | --- | --- | --- |
| Chadi et al. (2014)  *Incisional Negative Pressure Wound Therapy Decreases the Frequency of Postoperative Perineal Surgical Site Infections: A Cohort Study* | Retrospective cohort Study  Rectal cancers, anal malignancies and IBD | 59 | iNPWT device (KCI, San Antonio, TX)  -125 mmHg  5 days | Incidence of SSI  Lengh of stay | pNPWT group showed significantly lower SSI rates (14.8% vs. 40.6%, p = 0.04) |
| Chung et al. (2014)  *Incisional negative pressure therapy reduces perineal superficial wound infections following abdominoperineal resection* | Retrospective Cohort | 22 | unknown | Incidence of SSI | Lower SSI incidence in pNPWT group (9.1% vs. 41.7%, p = 0.012) |
| Van der Walk et al. (2017)  *Incisional Negative-Pressure Wound Therapy for Perineal Wounds After Abdominoperineal Resection for Rectal Cancer, a Pilot Study* | Prospective Pilot Study with historical control group  Rectal cancer & IBD | 10 | PICO system (Smith & Nephew, London, United Kingdom)  -80 mmHg  7 days | Incidence of SSI, wound dehiscence and complications | No significant difference in complications rates (70% vs. 60%) |
| Wiegering et al. (2017)  *Impact of incisional negative pressure wound therapy on perineal wound healing after abdominoperineal rectum extirpation* | Prospective Case Serie  Rectal cancer | 6 | iNPWT system (Prevena™ Incision Management System, KCI, Wiesbaden, Germany)  -100 mmHg  5 to 12 days | Incidence of wound dehiscence, SSIs | Reported 16.7% incidence of wound dehiscence in pNPWT group |
| Sumrien et al. (2016)  *The use of a negative pressure wound management system in perineal wound closure after extralevator abdominoperineal excision (ELAPE) for low rectal cancer* | Prospective Pilot Study  Rectal cancer | 32 | Unknown machine type  -125 mmHg  5 days | Incidence of wound dehiscence | Lower rate of wound dehiscence with pNPWT (9.4% vs. 40%, p = 0.01) |
| Kaneko et al. (2021)  *Incisional negative pressure wound therapy to reduce perineal wound infection after abdominoperineal resection* | Retrospective Study  Rectal cancer, anal cancer, IBD, faecal incontinence | 146 | ACTIV.A.C. Therapy System (KCI, San Antonio, Texas)  -125 mmHg  5 days | Wound healing outcomes, SSIs | Reduced SSI rates and improved healing outcomes in the pNPWT group |
| Salmenkylä et al. (2022)  *Case control study investigating the clinical utility of NPWT in the perineal region following abdominoperineal resection for rectal adenocarcinoma: a single center study* | Prospective Pilot Study  Rectal cancer | 21 | pNPWT (Avelle®, ConvaTech™)  -80 mmHg  7 days | Incidence of SSI, wound dehiscence | No significant reduction in SSIs; pNPWT device adherence problems were common |
| Rather et al. (2023)  *Closed Incisional Negative Pressure Therapy Reduces Perineal Wound Complications After Abdominoperineal Resection* | retrospective cohort study  Rectal cancer, anal cancer & IBD | 45 | iNPWT system (Prevena Plus™ Incision Management System, KCI)  -125 mmHg  4 to 7 days | Incidence of SSI, wound dehiscence | No significant reduction in SSIs; pNPWT device adherence problems were common |
