# Supplementary material for: Evaluating negative-pressure wound therapy after abdominoperineal resection: a systematic review of efficacy and technical variability
Source: Tech Coloproctol. 2025 Sep 23;29(1):168. doi: 10.1007/s10151-025-03212-5 (PMC12457546; doi:10.1007/s10151-025-03212-5)
Supplement: Supplementary file 2 — Supplementary file2 (DOCX 21 KB) [file 10151_2025_3212_MOESM2_ESM.docx]

Table 2. Postoperative Outcomes and Complications

| Study | n | SSI, n (%) | Wound dehiscence, n (%) | Intra-abdominal abscess, n (%) | ED visit, n (%) | Reoperation, n (%) | Length of stay (days) | Comorbidities (%, n) | Pre-op radiation (%, n) | Time to wound closure (days) | Device-related complications (%, n) |
| --- | --- | --- | --- | --- | --- | --- | --- | --- | --- | --- | --- |
| Chadi et al. (2014) | 27 | 4 (14.8%) | Not reported | 2 (7.4%) | 0 (0%) | Not reported | 11 | 15% diabetes | 59% | Not reported | Not reported |
| Chung et al. (2014) | 22 | 2 (9.1%) | Not reported | Not reported | Not reported | Not reported | Not reported | 20% obesity | Not reported | Not reported | Not reported |
| Van der Walk et al. (2017) | 10 | 7 (70%) | Not reported | Not reported | Not reported | Not reported | Not reported | 30% diabetes | Not reported | Not reported | Not reported |
| Wiegering et al. (2017) | 6 | Not reported | 1 (16.7%) | Not reported | Not reported | Not reported | Not reported | Not reported | Not reported | Not reported | Not reported |
| Sumrien et al. (2018) | 20 | Not reported | 2 (9.4%) | Not reported | Not reported | Not reported | Not reported | Not reported | Not reported | Not reported | Not reported |
| Kaneko et al. (2021) | 146 | 35 (24.0%) overallI  NPWT: 4 (7.8%)  Control: 31 (32.6%) | INPWT: 2 (3.9%) Control: 9 (9.5%) | Not reported | Not reported | Not reported | INPWT: 27 (11–156) Control: 26 (11–105) | Diabetes: INPWT 9.8%, Control 14.7% | INPWT: 13.7% (7/51)  Control: 13.7% (13/95) | Not reported | 5/52 (9.6%) skin blisters (1 discontinuation & excluded) |
| Salmenkylä et al. (2022) | 21 | 2 (10%) | 7 (33%)  2 seroma (10%) | Not reported | Not reported | Not reported | 15 | Not reported | 12 (57%) | Not reported | Device failure in 62% of cases |
| Rather et al. (2023) | 45 | 10 (32.3%) | Not reported | Not reported | Not reported | 13 (29%) | Not reported | Not reported | 18 (58.1%) | Not reported | Not reported |
