# Supplementary material for: Evaluating negative-pressure wound therapy after abdominoperineal resection: a systematic review of efficacy and technical variability
Source: Tech Coloproctol. 2025 Sep 23;29(1):168. doi: 10.1007/s10151-025-03212-5 (PMC12457546; doi:10.1007/s10151-025-03212-5)
Supplement: Supplementary file 3 — Supplementary file3 (DOCX 23 KB) [file 10151_2025_3212_MOESM3_ESM.docx]

Table 3. Quality Assessment of Included Studies Using the Newcastle-Ottawa Scale (NOS)

| **Study (author & year)** | **Selection (0-4)** | **Comparability (0-2)** | **Outcome/Exposure (0-3)** | **Total (0-9)** |
| --- | --- | --- | --- | --- |
| **Chadi et al. (2014)** | ★★★★ | ★ | ★★★ | 8 |
| **Chung et al. (2014)** | ★★★ | ★ | ★★ | 6 |
| **van der Valk et al. (2017)** | ★★★ | ★ | ★★★ | 7 |
| **Wiegering et al. (2017)** | ★★ | ★ | ★★★ | 6 |
| **Sumrien et al. (2018)** | ★★★★ | ★★ | ★★★ | 9 |
| **Kaneko et al. (2021)** | ★★★★ | ★ | ★★★ | 8 |
| **Salmenkylä et al. (2022)** | ★★★ | ★ | ★★ | 6 |
| **Rather et al. (2023)** | ★★★★ | ★★ | ★★★ | 9 |

Justification as requested by reviewer, not to be published

| **Study** | **Selection (0–4)** | **Comparability (0–2)** | **Outcome (0–3)** | **Total Score (0–9)** |
| --- | --- | --- | --- | --- |
| **Chadi et al. (2014)** | 4 – Clear cohort, prospective, representative population | 1 – Adjusted for pre-op RT but not other comorbidities | 3 – Objective outcome, adequate follow-up, used medical records | 8 |
| **Chung et al. (2014)** | 3 – Retrospective, unclear representativeness | 1 – Basic group comparison, limited adjustment | 2 – No info on follow-up duration, but objective outcome | 6 |
| **van der Valk et al. (2017)** | 3 – Small pilot, clearly defined but limited generalizability | 1 – Minimal adjustment for confounders | 3 – Clinical outcomes reported, short-term follow-up | 7 |
| **Wiegering et al. (2017)** | 2 – Very small sample, unclear representativeness | 1 – Limited baseline data | 3 – Reported dehiscence, based on clinical observation | 6 |
| **Sumrien et al. (2016)** | 4 – Clear eligibility, moderate cohort size | 2 – Reported on comorbidities and RT exposure | 3 – Adequate follow-up, wound outcome defined | 9 |
| **Kaneko et al. (2021)** | 4 – Large retrospective cohort | 1 – Descriptive stats only | 3 – Outcomes extracted from records, but unclear follow-up duration | 8 |
| **Salmenkylä et al. (2022)** | 3 – Case-control, matching unclear | 1 – Groups similar but no statistical adjustment | 2 – Device failure noted, but short follow-up | 6 |
| **Rather et al. (2023)** | 4 – Well described case-control population | 2 – Adjusted for major confounders | 3 – SSI and VAC use tracked and documented | 9 |
